# Effectiveness and cost-effectiveness of interventions to increase knowledge and awareness of attention deficit hyperactivity disorder: a systematic review

**DOI:** 10.1007/s00787-025-02646-4

**Published:** 2025-01-26

**Authors:** Kaitlyn McKenna, Sithara Wanni Arachchige Dona, Lisa Gold, Tim J. Silk, Ha N. D. Le

**Affiliations:** 1 Deakin Health Economics, School of Health and Social Development, Faculty of Health, Institute for Health Transformation, Deakin University, Geelong, VIC Australia; 2 Centre for Social and Early Emotional Development and School of Psychology, Deakin University, Geelong, VIC Australia

**Keywords:** ADHD, Economic evaluation, Knowledge

## Abstract

Various interventions, including caregiver education, psychoeducation, teacher and clinician training and behavioral management embedded with education, are available to enhance awareness and knowledge among caregivers, teachers, and clinicians. This review synthesizes evidence on the effectiveness and cost-effectiveness of interventions to increase ADHD awareness and knowledge for caregivers, clinicians, and teachers. Peer-reviewed literature was identified through the systematic searches of six databases: MEDLINE Complete, APA PsycInfo, CINAHL Complete, ERIC, Global Health and EconLit. Two independent reviewers completed a two-stage screening process and quality assessment. Results are presented as a narrative synthesis. Economic evaluation study cost data were converted for comparability. Of the 5963 results, 28 studies were included. Most interventions were found to be effective in increasing the awareness and knowledge of ADHD among caregivers, clinicians, and teachers. Clinician knowledge and capacity to assess, diagnose and treat ADHD were improved. Of the economic evaluation studies, interventions were promising in parent education, and behavioral management and psychosocial therapy interventions were found to be cost-effective. The review provides important insights about effective and cost-effective interventions to increase ADHD awareness and knowledge. These insights are critical to effectively plan services/interventions for families and children with ADHD. Further research in economic evaluation of interventions is needed to assist policy-decision making.

**Supplementary Information:**

The online version contains supplementary material available at 10.1007/s00787-025-02646-4.

## Introduction

Knowledge and awareness of attention-deficit/hyperactivity disorder (ADHD) is important to enable access to early and accurate diagnosis, and treatment, which is essential for improving the quality of life of children and their families [1–3]. Previously synthesized findings [4–6] demonstrate that a lack of awareness and knowledge of ADHD among caregivers, clinicians, and teachers is a key barrier to access and utilization of services. Evidence suggests that there are ADHD knowledge gaps among clinicians [3] and teachers [7, 8], identifying a need for specific ADHD education among these groups to improve service access and educational supports for children [7]. Caregivers of children with ADHD symptoms may be unaware that their child may meet an ADHD assessment criteria, be reluctant, or unable to access necessary health services due to systemic-structural barriers [9], such as stigma around mental health, lack of ADHD literacy, and limited access to specialized assessment [6]. Positive effects of interventions on caregiver ADHD knowledge and skills have been demonstrated; digital health interventions can significantly improve caregiver ADHD and mental health literacy [10]. Psychoeducation for parents can result in improved awareness and knowledge among caregivers, and improvements in child behavior, family functioning, and treatment outcomes [11, 12]. In addition to caregiver knowledge, two recent systematic reviews [5, 6] found that a lack of awareness, recognition, and knowledge of ADHD among clinicians can result in reduced confidence in recognizing and treating ADHD in patients within primary care settings [5, 6]. Specific ADHD training programs are important to ensure that clinicians can work effectively and support families to manage ADHD [13, 14]. There have been no previous reviews of the evidence for interventions to improve ADHD awareness or knowledge among clinicians.

Teachers also play a crucial role in referring children with ADHD to clinical care [15]. Research demonstrates that a lack of knowledge and experience with ADHD among teachers can prevent support for children with ADHD symptoms, particularly among teaching assistants [6, 16, 17]. Improving teachers' knowledge about the condition, their attitude towards children, and interdisciplinary collaboration can result in positive outcomes for children with ADHD and increase access to support [18]. Training interventions have been developed to improve knowledge about ADHD among teachers to increase recognition of behavioral difficulties and to support children in the classroom [19]. A recent systematic review and meta-analysis investigating the effects of ADHD training for teachers found that interventions can be effective in improving teachers' knowledge about ADHD. However, improvements in knowledge were not retained at follow-up, but still remained above pre-intervention levels, and there is inconsistent evidence to suggest that teacher training reduces pupil ADHD-type behaviors [20]. This review only included studies with a controlled trial published until 2020 and focused on training for primary or secondary school teachers [20]. The exclusion of other study designs, and study populations, such as special education teachers, may have resulted in missing relevant information on interventions to increase teacher knowledge of ADHD. Therefore, we seek to include all teacher types, and to update this review with recently published literature on teacher intervention studies.

Given that population health needs always exceed available healthcare resources, it is crucial to assess the cost and cost-effectiveness of interventions, in addition to their effectiveness. Research on the cost-effectiveness of ADHD interventions aiming to improve ADHD understanding is scarce. Previous economic evaluation studies have focused on the cost-effectiveness of ADHD treatment for children [21]. This systematic review aims to synthesize all the existing evidence on the effectiveness and cost-effectiveness of interventions for caregivers, clinicians, and teachers on awareness and knowledge of ADHD.

In this review, ‘awareness’ refers to the general understanding that ADHD is a condition that often requires diagnosis, treatment, and support [22]. ‘Knowledge’ refers to having an educated, factual understanding of ADHD [22].

## Methodology

This systematic review followed PRISMA guidelines 2020 [23] and was registered with PROSPERO (CRD42023448044) in August 2023 [24].

### Search strategy

The search strategy was developed by the primary reviewer (KM) through a preliminary search and consultation with the review team and a librarian. A comprehensive search was conducted in EBSCOhost databases (MEDLINE Complete, APA PsycInfo, CINAHL Complete, ERIC, Global Health and EconLit) to search for literature published until 8th August 2023 and updated on 16th February 2024. Multiple search terms were used for three concepts: (1) attention-deficit/hyperactivity disorder, (2) economic evaluation, (3) intervention and awareness (Appendix A). A Google Scholar search was also conducted to identify any relevant studies (Appendix A).

### Inclusion and exclusion criteria

Studies were included if they were (1) peer-reviewed publications in English, (2) explored the effectiveness or cost-effectiveness of interventions for ADHD awareness and knowledge for caregivers, clinicians, or teachers, (3) primary quantitative studies. Studies on intervention effectiveness were included if the primary outcome measured was awareness, knowledge or evidence-based practices outcomes. Due to limited economic evaluation literature, we included interventions with educational/knowledge components, but with any outcome measure (not restricted to measuring awareness/knowledge changes). Studies were excluded if they (1) did not meet the above criteria, (2) only used qualitative data collection methods or were a secondary study (review). Secondary studies (i.e., reviews) identified from the search were hand-searched to exclude any primary effectiveness studies included in those reviews to avoid repeating results.

### Study selection process and data extraction

All studies retrieved from the search were exported to Endnote [25] and then imported into Covidence [26]. A pilot screening was conducted with a random sample of one hundred studies to ensure agreement of the inclusion criteria. The studies were screened independently by two reviewers (KM and SWAD) through a two-stage process: (1) title and abstract and (2) full-text. Any disagreement among the two reviewers was discussed until a consensus was reached, with resolution through a third reviewer (HL or TS) if necessary. The primary reviewer completed the data extraction, which was cross-checked by the second reviewer. For effectiveness studies, the extracted information described the author, year, country, study design, target population and sample, ADHD status, intervention type and description, assessment tools, primary outcome, and results. For studies reporting cost or cost-effectiveness, additional data were extracted on study perspective, cost year, time horizon, and discount rate.

### Quality assessment

The methodological quality of the included studies was independently assessed by the two reviewers using (1) The National Heart, Lung, and Blood Institute (NHLBI) quality assessment tools [27] for Controlled Intervention Studies, Before-After (Pre-Post) Studies With No Control Group, and Observational Cohort and Cross-Sectional Studies, because of its rigorous criteria, allowing a consistent assessment of various study designs [27] and its wide use [28–32], (2) Mixed Methods Appraisal Tool [33] for Quantitative non-randomized studies (pre-post with non-randomized control group), as NHLBI tool for controlled studies primarily focuses on randomized controls, whereas the MMAT provides criteria for evaluating non-randomized studies [33] and (3) Drummond’s checklist for economic evaluation studies [34]. The description of each tool and the applicable questions used are outlined in Appendix B, and the scoring technique and quality assessment results tables are described in Appendix C. Disagreements were resolved through discussion until a consensus was reached.

### Analysis

A narrative synthesis approach was used to synthesize study findings [35–37] whereby study characteristics were tabulated and studies were subgrouped by the intervention target population (i.e., caregivers, clinicians, teachers) and the primary outcomes measured; the findings were synthesized through the frequency and type of outcomes reported [35]. A meta-analysis was not conducted due to the heterogeneity among the studies’ methodology and outcome measures. When reporting the findings from the economic studies, all costs were converted into 2024 purchasing power parity (PPP) estimates (international dollars) using a standard conversion tool [38] to allow comparability across countries [39].

## Results

### Study characteristics

**Fig. 1:**
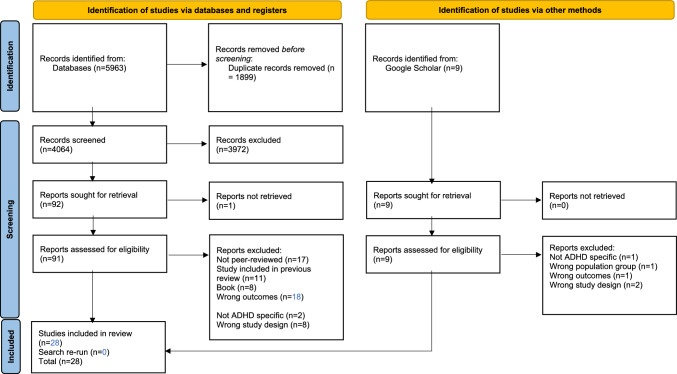
PRISMA diagram

The database search yielded 5963 studies, of which 24 were included in the analysis. Google Scholar search identified a further four studies, resulting in 28 studies included in the analysis. The updated search found no further studies to be included (Fig. 1). There was an inter-rater agreement of 85% among the two reviewers for the full-text screening. Included studies (*n* = 28) represented a wide range of countries; fifteen studies from North America [United States (*n* = 14) and Canada (*n* = 1)], eight from Europe [United Kingdom (*n* = 5), Greece (*n* = 1), Spain (*n* = 1), Sweden (*n* = 1),], two from Asia [India (*n* = 1), Saudi Arabia (*n* = 1)], two from Africa [Egypt (*n* = 2)], and one from Oceania [Australia (*n* = 1)] (Table 1). Twenty-four studies evaluated or reported the effectiveness of an intervention, of which nine utilized a randomized control trial design, three used a quantitative non-randomized design (pre/post-test) with control group, thirteen studies were a before-after (pre-post) with no control group design, and one was a cross-sectional study. The four economic studies were cost-effectiveness analyses.

**Table 1:** Effectiveness study characteristics and key findings

Author and year	Country	Study design	Target population	Sample	ADHD status	Comparison	Intervention type	Intervention	Assessment tool (relevant)	Primary outcome	Results
Caregivers/parents											
Dixon et al. (2023)	United States	Pre/post-test	Caregivers	68 parents/caregivers of children (14–15 years old)	At-risk of ADHD via parent reporting, or diagnosed ADHD	None	Parent education	× 1 2-h workshops. Presentation and QA. Content: ADHD prevalence, diagnostic criteria, evaluation, myths, evidence-based treatments	ADHD Beliefs and Attitudes Scale	Parent knowledge	Significant knowledge and belief changes regarding effectiveness of medication treatments, willingness to accept physician treatment recommendations, and were less likely to accept non-evidence-based treatments. Improvements in beliefs in behavior management knowledge pre and post mean (SD) 6.04 (0.71) vs. 6.20 (0.75), beliefs of medication as effective 4.46 (0.95) vs. 5.22 (1.13)
DuPaul et al. (2018)	United States	RCT	Caregivers	47 parents of children (3–5 years 11 months old) Face to face (F2F) (*n* = 16) Online (*n* = 15)	DSMV criteria for one of three ADHD presentations	Waitlist control (*n* = 16)	Behavioral Parent Training	× 10 F2F sessions. × 10 online sessions. Lecture, group discussion, role-play, quizzes, and videos. Content: ADHD introduction, routine, problem solving, prevention/instructive/response strategies, academic success, communication	Study-own test of parent knowledge behavioral techniques and ADHD information	Parent engagement, parent stress and child behavior	Parents in the F2F group (*p* < 0.01; Cohen’s *d* = 1.49) and online group (*p* < 0.05; Cohen’s *d* = 0.74) demonstrated higher knowledge of behavioral techniques and ADHD information scores at post-test than parents in the control group (no statistical difference between the two treatment groups)
Gerdes et al. (2021)	United States	RCT (Pre/post-test (randomized control))	Caregivers	58 Latinx families with children (5–13 years old) Culturally adapted treatment (CAT) (*n* = 31)	ADHD diagnosis as part of study	EBT comparison (*n* = 27)	Psychoeducation	CAT: × 8 2-h parent management training sessions, culturally adapted. Individualized DRC with weekly parental involvement and two home visits EBT: × 8 2-h parent management training sessions, individualized classroom intervention of a DRC	ADHD knowledge measure	Parent knowledge	Parents who received the CAT demonstrated knowledge improvement by correctly identifying more ADHD symptoms, missed fewer symptoms, and had higher total ADHD symptoms score post-test, compared to pre-test EBT showed no significant findings for mothers, whereby fathers experienced decrease in symptom identification
Odom (1996)	United States	RCT (pre/post-test (randomized control))	Caregivers	25 low SES female parents with male children (5–11 years old) (intervention *n* = 13)	ADHD diagnosis as part of study	Non-educational group (*n* = 12)	Behavioral Parent Training	× 5 60–90-min sessions (shortened version of Barkley's parenting program). Content: general ADHD information and stimulant treatments, child misbehavior, parental positive reinforcement, behavior management (time-out), and problem solving	Parenting Sense of Competence Scale ADHD Knowledge and Opinion Scale	Caregiver knowledge Caregiver self-esteem and competence	Overall knowledge (pre- 9.0 (1.82) to post- 10.0 (1.24)), medication willingness (pre- 27.8 (1.68) to post- 29.0 (1.42)), and competence (pre- 11.4 (1.83) to post- 12.3 (.99)) increased in the educational group, with a slight decrease in counselling willingness. Non-educational group knowledge (pre- 9.7 (1.8) to post- 9.3 (1.6))
Jimenez et al. (2022)	Spain	RCT	Caregivers	48 Parents and their children (6–12 years old) Intervention (*n* = 16)	ADHD DSM5 diagnosis	Control (*n* = 32)	Psychoeducation	× 9 90-min group sessions. Program based on researcher’s experience and Barkley's Manual. Content: Role of play in relationships, overview of ADHD, Barkley’s 4 factor model learning and behavior modification techniques, care, and collaboration to increase behaviors, reduce inappropriate behaviors, limits, and ADHD and school	Parent and teacher ratings of ADHD symptoms ADHD knowledge questionnaire Study-own knowledge of drug treatment questionnaire	Parent knowledge ADHD symptoms	Post-test data showed statistically significant difference in the intervention group in knowledge of ADHD ((SD) 11.5 (7.3) 17.3 (5.5) *p* = 0.008), and knowledge of treatment drugs ((SD) 4.5 (1.9) 6.1 (1.0) *p* = 0.005)
Ryan et al. (2015)	England/United Kingdom	Pre/post-test	Caregivers	172 parents/ caregivers of a young person (4–18 years old)	Confirmed or suspected ADHD diagnosis	None	Parent education	ADHD & You website containing principles and management techniques for ADHD. Four sections target parents, healthcare professionals, individuals with ADHD, and education staff, links to external resources and downloadable resources for each group	Knowledge questionnaire (adapted from AKOS-R)	Parent knowledge	Significant increase in ADHD knowledge (pre/post-test difference 0.63 (1.46)) in participants who accessed the website. Post-test scores demonstrated moderately significant difference in knowledge between website users and non-users (post-scores −2.473, *p* = 0.013)
Shata et al. (2014)	Egypt	Pre/post-test	Caregivers	50 mothers presenting at a health clinic	ADHD diagnosis	None	Psychoeducation	× 8 45–60-min weekly group sessions Illustrations, vignettes, role playing, brainstorming, and group discussions. Content: ADHD overview, child behavior, positive parenting practices, child learning problems, stress management and problem solving	Parental ADHD-related knowledge questionnaire	Caregiver knowledge and skills	Mothers ADHD knowledge improved significantly at both post-test 1 and 2 compared to pre-test (*X*2 = 89.63, *P* 0.001, *X* ± SD = 10.78 ± 3.33 pre-test, 17.32 ± 2.37 post-test 1, and 17.24 ± 2.33 post-test 2). Improvement in parenting practices decreased at post-test 2, compared with post-test 1, however, remained significantly better than pre-test
*Clinicians*											
French et al. (2020)	England	RCT	Clinicians	221 general practitioners Intervention (*n* = 111)	None	Control (*n* = 110)	Web-based psychoeducation program	Web-based resource consisting of × 2 20-min modules. Module 1: understanding ADHD (prevalence, symptoms, misconceptions). Module 2: Role of the GP, ADHD diagnosis, treatment and care pathways in the UK, and an ADHD toolkit Control group: 30-min video about mental health	KADDS Questionnaire (adapted) GPs’ understanding of the ADHD questionnaire Awareness of GPs of the ADHD Questionnaire	GP Knowledge	The intervention group showed significantly more knowledge of ADHD (F1,106 = 117.5, *p* < 0.001) retained at two-week follow up and a significant reduction in ADHD misconceptions (F1,106 = 4.20, *p* = 0.04), and increase in confidence (F1,106 = 182.8, *p* < 0.001) compared to the control group (retained at follow up) (F1,106 = 110.08, *p* <0 .001). High levels of self-rated confidence were associated with higher scores of ADHD knowledge (*r* = 0.473, *n* = 109, and *p* <0.001)
Loskutova et al. (2021)	United States	Pre/post-test	Clinicians	97 physicians and providers from 6 primary care practices	None	None	Web-based toolkit	Web-based AAFP Adult ADHD Toolkit Content: general ADHD knowledge, assessment and diagnosis, treatment, and management, reducing risk, and FAQ	Weekly provider surveys Web analytics data Knowledge and confidence were assessed using predefined domains	Provider knowledge and confidence	Significant improvements in toolkit user's knowledge were in monitoring for treatment effects, side effects and outcomes (midpoint 3.6 vs baseline 3.0; *p* = 0.004), resources for patients and clinicians about ADHD (midpoint 3.3 vs baseline 2.9; *p* = 0.03), and management of ADHD with comorbidity’ (midpoint 3.2 versus baseline 2.7; *p* = 0.01). Toolkit users reported higher confidence levels than non-users in mental health interview techniques (3.5 vs 3.0; *p* = 0.03), treatment choices for ADHD and other comorbidities (3.2 vs 2.3; *p* < 0.001)
Ward et al. (1999)	Canada	Pre/post-test	Clinicians	100 physicians	None	None	Training	× 1 day-course, didactic presentation, and case discussion held in groups with physician/psychiatrist assistance. Content: overview of ADHD, screening, diagnosis, treatment, local resources, and case-based discussions	Three-part needs assessment: 42 item knowledge survey Chart review	Physician knowledge	The post-test knowledge score (32.6) was statistically significantly higher than the pre-test knowledge score (27.1, *p* = 0.000). There was a 0.7 increase from pre to post-test for involvement measures, demonstrating physicians were providing more services to patients (3.0 (SD = 1.18) to 3.7 (SD = 0.9), *p* = 0.000). Chart data for physicians’ showed frequency in carrying out procedures (diagnosis making, treatment planning and follow-up behaviors) increased
Baum et al. (2019)	United States	Pre/post-test	Clinicians	29 hospital/school/community pediatric primary care practices	None	None	State-wide learning collaborative/quality improvement	× 4 interactive on-site training sessions on addressing mental health, care model, and communication. × 11 online learning modules	Study-own clinician confidence survey	Confidence mental health-related visits prescribing practices	Clinician confidence scores increased by 20% (95% CI 15–25%) from 2.92 at baseline, to 3.55 at post-test. Office visits for a mental health condition rose from 6.65% to 9% post-test, driven by detection and treatment of ADHD. Rates of ADHD medication prescribed increase (by 0.12 percentage points per month (ci = 0.02 to 0.22, *p* = 0.022)
Epstein et al. (2010)	United States	Pre/post-test	Clinicians	38 pediatricians across 14 primary care practices	None	None	Quality Improvement	× 2 1.5-h didactic sessions, each followed with a 1-h office-based training session (intervention total of × 4 sessions, 5-h). Focused on American Academy of Pediatrics ADHD guideline recommendations	Vanderbilt ADHD Rating Scales Chart reviews	Quality Improvement	Chart reviews of 214 children with ADHD; physicians demonstrated substantial post-training improvement in evidence-based ADHD practice behaviors, including written care management plans, and follow up after medication initiation. Short term practice improvements were maintained for 24 months following the intervention; whereby all ADHD practice behaviors continued to be significantly improved from pre-test
Newcomb et al. (2022)	Australia	Pre/post-test	Clinicians	65 primary care providers, including 43 general practitioners	None	None	Collaborative model	ECHO Model: education and case-based discussion between pediatricians and GPs × 10 90-min weekly video conference sessions. Panelists presented aspects of ADHD management, clinicians presented cases to peers, and discussion of evidence-based recommendations	ECHO self-efficacy survey	GP's Self-efficacy in management of pediatric ADHD	Survey results showed a significant difference in the pre- and post-test in all domains. The maximum effect was demonstrated in the stimulant prescription, adjusting doses, weaning, and changing from short to long-acting stimulants, and assessment of anxiety
Pfiffner et al. (2023)	United States	Pre/post-test	Clinicians	School Mental Health Providers (SMHP) at district K-5 elementary schools (*n* = 8) Parents Teachers Students	Elevated ratings of ADHD symptoms /impairment	None	Evidence-based psychosocial intervention	Adapted version of the Collaborative Life Skills Program, conducted in a web-based remote method. Contained skill modules, handouts, video clips, tools, and coaching SMHP were trained, and delivered the intervention: × 8 parent, and × 8 45-min child group sessions. × 2 60-min group teacher sessions, and 1–2 parent/teacher/student meetings	ADHD and ODD symptoms Child and symptom inventory Clinical Global Impressions (CGI) Scale Improvement Children’s Strengths and Difficulties Questionnaire	Clinician outcomes Child symptoms	SMHPs average ratings improved significantly from pre- to post-intervention for perceived knowledge of skills and confidence in delivering skills across program components (parent, child, and teacher)
*Teachers*											
Alshehri et al. (2020)	Saudi Arabia	RCT	Teachers	100 male teachers (government/private male primary schools) Intervention (*n* = 50)	None	Waitlist control (*n* = 50)	Knowledge improvement program	× 2 day F2F: × 1 one-hour lecture on ADHD overview, prevalence, cause, symptoms, treatment approaches, open discussion, and second day workshop about ADHD toolkit Control: Educational materials after the third assessment	Self-administered questionnaire (complied by Awadalla et al., based on Kos)	Teacher's knowledge	Knowledge in intervention group significantly improved post-test with slight decline at follow-up (3 months), remained higher than control. Control showed statistically significant knowledge improvement in progress of disease and treatment. Participants in intervention group with higher qualification were associated with significant knowledge retainment (*p* < 0.001 and *p* = 0.050, respectively)
Awadalla et al. (2016)	Egypt	Cross-sectional	Teachers	39 principal teachers from public and private primary schools	None	None	Knowledge/capacity improvement program	× 2 day, F2F workshop, × 2 h each. Presentation on ADHD definition, symptoms, management in classroom, role of teachers in early detection	Self-administered questionnaire (compiled in study, based on Kos)	Teacher's knowledge to improve early detection of ADHD	Post-test showed significant improvement in aspects of disease knowledge (from 69.2% for disease progress to 94.9%), significantly higher than pre-test scores. Significantly higher knowledge among 1–4 years of teaching than those 5 + years. Mean score (SD) for knowledge aspect (pre-test vs post-test 8.26 (6.28) vs 16.92 (4.09))
Bradley-Klug et al. (1997)	United States	Pre/post-test	Teachers	Level 1: 169 school personnel from 57 school districts. Level 2 & 3: Direct consultation for 169 students, 492 parents	Students with behavioral difficulties related to ADHD	None	School-based collaborative consultation model	Three levels: 1) × 2 day in-service program on school-based ADHD assessment/ identification, behavior management, medication monitoring, social skills, problem solving. 2) 30 h of on-site consultation (system or individual level). 3) Follow up consultation, further training	Knowledge test of ADHD (Anastopoulos, Shelton & DuPaul)	Participation Personnel and students reached Services used	Post-test knowledge of ADHD demonstrated a statistically significant improvement from pre-test (*t* = −17.21, *p* < 0.01) of 18%; a mean of 75% at pre-test, to 93% post-test among school personnel from 57 school districts
Giannopoulou et al. (2017)	Greece	Pre/post-test	Teachers	143 teachers Group 1: nursery/primary school teachers in state schools (*n* = 68), group 2: teachers of postgraduate training in special education (*n* = 75)	None	None	Training seminar	Group 1: Half-day, 5 h Group 2: × 2 day, 18 h Both groups attended educational seminars., also included scenarios and situation techniques. Topics: general ADHD knowledge, impact on learning, teacher strategies, treatment approaches, screening instruments for teachers	Study-own self-report ADHD Knowledge Questionnaire (ADHD-KQ)	Teacher knowledge	Pre- and post-test knowledge scores; difference of mean score of 6.96 ± 4.83 for the Group 1, and of 7.91 ± 4.94 for the Group 2, were statistically highly significant (p < 0.001) All ADHD-KQ sub-scales indicated significantly improved (p < 0.001) teacher's knowledge of ADHD in all domains Teachers in group 2 showed significantly higher pre-post difference compared to teachers in group 1
Jones and Chronis-Tuscano (2008)	United States	RCT	Teachers	142 teachers from 6 elementary schools In-service group (*n* = 74)	None	Waitlist control (*n* = 68)	In-service training	In-service training in presentation format, with handouts on ADHD and daily report cards. Topics: general ADHD information, evidence-based treatment for ADHD, classroom behavioral management strategies	Study-own ADHD knowledge questionnaire Teacher Use of Classroom Behavior Management Strategies scale	Teacher knowledge Use of evidence-based strategies	The immediate in-service group demonstrated significantly improved (small to medium size effects) knowledge of ADHD from pre- (19.5 (1.9)) to post-test (20.4 (1.8)), to a greater extent than the control (pre- 18.9 (2.1)) to post-test (19.0 (2.4)). Significant increase in use of behavior management strategies from pre- to post-test, control did not
Monteiro (2023)	United States	Pre/post-test	Teachers (preservice teachers—undergraduate students)	71 pre-service teachers at a university	None	None	Training	× 1 online webinar; 3 videos, total of 1.2-h. Topics: 1) symptoms, 2) assessment and diagnosis of ADHD, and 3) evidence-based interventions (behavioral, cognitive, and educational)	Knowledge of Attention Deficit Disorders Scale (KADDS) Teacher Self‐Efficacy Scale Usage ratings profile‐web resource	Teacher knowledge and self-efficacy	Pre-service teachers demonstrated significant increase in knowledge of ADHD identification and interventions from pre- (M = 14.39, SD = 3.89) and post-test (M = 24.35, SD = 8.03). Training resulted in misconception scores changing unexpectedly. Self-efficacy significantly increased (small increase) across participants from pre- (M = 6.67, SD = 1.36) to post-test (M = 7.23, SD = 1.20)
Sayal et al. (2006)	England /United Kingdom	Pre/post-test	Teachers	96 teachers of 6 primary schools 2672 students (4–11 years old)	ADHD screening using Strengths and Difficulties Questionnaire	None	Educational intervention	Pilot study: × 1 45-min educational session about ADHD, contained video clips, handouts/resources. Content: overview of ADHD, presents at school, role as risk factor, outcomes, symptoms, diagnosis and comorbidity, medication, and classroom management strategies	DSM-IV ADHD criteria (teacher recognition)	Recognition of ADHD	Teachers’ ability to detect probable ADHD in children improved; at baseline, 3.2% having probable ADHD and 8.2% possible ADHD. Post-test, the rate of probable ADHD increased to 4.1%. Nearly half of the pupils regarded as probable, were previously identified as possible ADHD. 92% agreed that they were able to recognize ADHD symptoms. 87% felt better informed about ADHD and 81% felt more confident about recognizing childhood ADHD
Srivastava et al. (2015)	India	Pre/post-test (non-randomized control)	Teachers	79 primary school teachers Intervention (*n* = 38)	None	Control (*n* = 41)	Training	× 4 consecutive days, 2.5-h sessions. Used presentation, video clips and small discussion. Topics: rights-based approaches to inclusive education, and facts, behaviors, and teaching methods for each condition. Addressed ADHD, dyslexia, intellectual disability, and ASD	Multidimensional Attitudes Toward Inclusive Education Scale (adapted) Study-own special education needs/teacher methods knowledge questionnaire	Teacher attitude and knowledge	Knowledge about ADHD in the experimental group from pre- (3.09 .36) to post-test (3.09 .38) was not statistically significant. No increase was observed in the control group. The experimental group identified the description of the vignette more correctly and less incorrectly at post-test, compared to the control group (for ADHD), indicating some form of knowledge improvement
White et al. (2011)	United States	RCT (Pre/post-test (randomized control))	Teachers	137 teachers from 5 elementary schools	None	Comparison (*n* = 11)	Training	Workshop part of annual staff training program. × 1 2-h workshop of 60 presentation slides about Tourette syndrome, OCD and ADHD. Covered overview (history, prevalence), treatment, classroom strategies and methods	Study-own 27-item knowledge test	Teacher knowledge	There was a significant increase of teacher knowledge (*t*(51) = −4.57, *p* < 0.001) of ADHD from pre-test (35 (23.72)) to post-test (52 (23.74) + 17%) compared to the comparison group pre- (33 (24.12)) to post-test (41 (20.23) + 8%)

### Methodological quality of included studies

Of the 22 studies that were assessed using the NHLBI checklists [27], most (59%) were rated as fair quality, and almost one third (27%) were rated as poor quality. Of the two studies that were assessed using the Mixed Methods Appraisal Tool [33], one was rated as high, and one as medium quality. The difference in quality ratings between the tools may be explained by the focus of the NHLBI critical appraisal on the internal validity of a study, whereby risk of bias due to methodological limitations was frequent: poor reporting of randomization techniques, lack of blinding, and poor reporting of sample size power. Three out of four economic evaluation studies met greater than 75% of quality criteria of Drummond’s 35-item checklist [34], indicating good quality (Appendix C).

## Effectiveness studies

### Caregiver

#### Overview

Interventions in seven studies provided ADHD knowledge to caregivers and parents [40–46] (Table 1). Two studies reported effect sizes ranging from small to medium [42] and medium to large [41] for changes in knowledge from pre-post intervention, however confidence intervals were not reported.

#### Caregiver knowledge/awareness

Seven studies assessed interventions that provided ADHD knowledge to parents and caregivers. Interventions ranged from a website resource, a single 2-h workshop to 5–10 weekly 1–2 h sessions. Studies reported improvements in caregivers’ overall/general ADHD knowledge [41, 43, 45, 46], ability to identify ADHD symptoms [42], understanding of behavioral management techniques [40, 41], knowledge of medication treatment [44], and medication willingness [43]. Caregivers demonstrated increased willingness to accept physician treatment recommendations and showed reduced likelihood of accepting non-evidence-based treatments [40]. DuPaul et al. [41] reported that both the face-to-face and online format had similar results.

### Clinician

#### Overview

Seven studies described interventions to increase knowledge of ADHD among clinicians [14, 47–52] (Table 1). The clinicians included across studies varied: pediatricians, both pediatricians and other health professionals (i.e., nurses, mental health specialists), general practitioners, and school mental health providers. Statistically significant increases in knowledge and evidence-based practices were reported in seven studies, with one study reporting large effect sizes [52].

#### Clinician knowledge/awareness

Three of the seven studies focused on interventions which addressed basic knowledge of ADHD among clinicians. Intervention types included a 1-day on-site course [14], and web-based approaches (toolkit and short modules) [49, 50]. These interventions were shown to be effective in improving clinicians’ knowledge on ADHD symptoms, behavioral management techniques, and effective communication with caregivers; all three provided general information about ADHD (i.e., ADHD symptoms, causes, prevalence, assessment and diagnosis, treatment, and behavioral management strategies) and knowledge on specific topics such as the role of clinicians, local resources, and care pathways [14, 49] These studies reported statistically significant knowledge increases post-intervention including a reduction in ADHD misconceptions [14, 49] and increased confidence in general practitioners’ knowledge of ADHD [49, 50].

#### Clinician ADHD capacity and evidenced-based practices

Four studies evaluated effectiveness of interventions targeting clinicians’ capacity and evidence-based practices for ADHD diagnosis, treatment, and management. Interventions varied from collaboration, quality improvement training, and psychosocial intervention, ranging from 4 to 10 sessions. The interventions effectively improved clinician’s capacity in ADHD diagnosis and management; ability to conduct evidence-based practices such as ADHD assessment [48], medication prescription/initiation and management of dosages [47, 51], written care management plans and follow up appointments [48] and improved knowledge of evidence-based practices for attention and behavior problems among school clinicians (delivery of a program to teachers (Cohen’s *d* −1.37, 95% CI (2.33, −0.36)) and parents (Cohen’s *d* −2.43, 95% CI (−4.08, −0.75) [52]. The quality improvement intervention delivered by Epstein et al. [48] demonstrated that practice improvements were maintained for 24 months following the intervention, which consisted of 5 h training; all ADHD practice behaviors continued to be significantly improved from what was reported at baseline.

### Teacher

#### Overview

Ten studies included teachers as the target sample for the intervention [53–62] (Table 1). The teaching level ranged from a majority of pre-school to elementary/primary school, middle/junior high school, with the ninth grade as the highest teaching grade. Two studies included graduate student teachers and preservice teachers (undergraduate students). Effect sizes for increases in knowledge were reported in three studies ranging from small to medium [57, 59, 62] and medium to large effects [62], however, confidence intervals were not reported.

#### Teacher knowledge/awareness

Interventions were training based, knowledge improvement programs, courses, and a consultative model. Duration ranged from single to multiple sessions of 45 min to two-and-a-half hours, half to 2-day training courses, and frequent consultation. Improvement in ADHD knowledge (symptoms, causes, prevalence, assessment and diagnosis, and treatment) and knowledge of school-based behavior management strategies among teachers were reported [53–60, 62]. Teachers’ capacity to identify ADHD symptoms in children improved [60] and special education teachers showed an increase in use of behavior management strategies with an identified child [57]. One study reported unexpected negative outcomes, whereby misconception scores increased from pre- to post-test [59]. This highlights the need for pre-intervention misconception assessments and follow-up reinforcement sessions. Srivastava, de Boer and Pijl [61] reported that ADHD knowledge increased without a statistically significant difference from pre- to post-intervention.

### Economic evaluation

#### Overview

Four studies conducted economic evaluations (cost-effectiveness analysis) on interventions for ADHD, including behavioral interventions [63, 64], parent training [65] and a psychosocial program with educational component [66] (Table 2). The methodology of the four studies varied: studies assessed different interventions; were conducted from either a combined health service provider and societal (*n* = 3) or payer (*n* = 1) perspective; time horizons ranged from 3 months to 6 months, with only one of the cost-effectiveness studies over 1 year which reported a discount rate of 3%; and reported outcomes using a mixture of generic and ADHD-specific measures. All four studies indicated promising and cost-effective results, depending on program intensity and delivery format.

**Table 2:** Economic evaluation study characteristic and key findings

Author	Country	Target population	Comparison group	Intervention type	Intervention	Study Design	Economic perspective	Cost year, discount and time horizon	Outcome measures	Cost component/cost items	Cost of the intervention	Results
Nystrand et al. (2019)	Sweden	Parents of children (5–12 years old) Interventions: Comet (*n* = 207) COPE (*n* = 202) Incredible Years (IY) (*n* = 122) Connect (*n* = 218) Bibliotherapy (*n* = 126)	Waitlist control (*n* = 159)	Behavioral programs	COPE: × 10 2–2.5 h weekly sessions, max. 25 parents. Discussion, modelling, roleplay, homework, self-monitoring. (social learning theory/attitudes/family systems) Connect × 10 1-h weekly group sessions of 12–14 parents. Teaching, roleplay, hand-outs (attachment theory) Comet: × 11 2.5-h weekly sessions of 10–12 parents. Teaching, roleplay, homework, video vignettes, handouts, individual meeting. (Webster-Stratton and Patterson’s, Barkley’s models) IY: × 12 2–2.5-h weekly sessions of 10–14 parents. Teaching, roleplay, group discussion, homework, modelling (cognitive social learning, self-efficacy) Bibliotherapy: self-help parent management booklet based on Comet	Cost-effectiveness	Payer perspective	2015 US$ Discount rate of ate of 3% per annum 0% and 6% in sensitivity analysis Until end of childhood (18 years old)	Eyberg Child Behavioral Inventory Swanson, Nolan, and Pelham Scale (SNAP-IV) Clinically Significant Reliable Change Index (CS/RCI)	Training costs Running costs	The total intervention costs ranged from $2335 ($2740) Bibliotherapy, to $163,865 ($192,266) Comet, $71,906 ($84,369) Connect, $76,888 ($90,214) COPE, and $135,414 ($158,884) IY The average total costs per child were $14 ($16) Bibliotherapy, $334 ($392) Connect, $477 ($524) COPE, $931 ($1092) Comet, and $1302 ($1528) IY	The IY program had the highest effect of averted DALYs (0.23 per participant), while Connect had the lowest (0.06) Bibliotherapy, Connect and COPE had lower accumulated net costs, and greater health benefits in terms of averted DALYs compared to the waitlist control The incremental net monetary benefits increased by up to 50% at times (Connect $3087 ($3622) per child at base case to $6790 ($7967) All five interventions were 100% likely to be cost-effective at a $80,000 ($93,866) WTP threshold per DALY and remained so when lowering the threshold to $15,000 ($17,600)
Sayal et al. (2016)	England	Parents of children (4–8 years old) Intervention Parent-only, and parent and teacher intervention, total follow-up: parents (*n* = 76), teachers (*n* = 169)	Control *n* = 72 (4 schools). Follow up: teachers (*n* = 52), parents (*n* = 37)	Behavioral program	Intervention based on the 1–2–3 Magic parenting program (Phelan). × 3 2-h sessions over consecutive weeks. Content: positive behavior strategies, management of difficult behaviors, and relationship with child. Supplemented with booklet and disc The combined intervention consisted of the program, with × 1 1.5-h group session for teachers. Addressed children’s needs, and reflection of teacher practices	Cost-effectiveness	National Health Service and personal social services (PSS) perspective Societal cost perspective	2012 UK£ No discount 6 months	Strengths and Difficulties Questionnaire (SDQ) Conners’ Rating Scale Malaise Inventory Acceptability feedback Client Service Receipt Inventory (CSRI) HRQoL- the EQ-5D-Y and the CHU9D for calculation QALYs	Running costs, service use and personal costs, and costs of productivity losses	The intervention costs of the parent-only was £90 ($178), and combined intervention was £107 ($211)	There was no effect of the parent-only (mean difference = −1.1, 95% CI −5.1, 2.9; *p* = 0.57), and combined intervention (mean difference = −2.1, 95% CI −6.4, 2.1; *p* = 0.31) on the ADHD index. All three groups showed improvement in mean EQ-5D-Y and CHU9D index values (not significant) The incremental costs of the parent-only and the combined interventions were £73 ($144) and £123 ($243), respectively. The incremental cost per one-point improvement was £29 ($57) parent-only, and £134 ($265) for the combined intervention Above a willingness-to-pay of £31 ($61) per one-point improvement in parent-rated ADHD index, parent-only program had the highest probability of cost-effectiveness
Sonuga-Barke et al. (2018)	United Kingdom	307 Parents with children (2 years 9 months to 4 years and 6 months old) New Forest Parenting Program (NFPP) (*n* = 134), Incredible Years (IY) (*n* = 131)	Treatment as usual (*n* = 42)	Parent training	NFPP (adapted): 12-week, 1.5 h sessions: individually tailored and home-delivered program with four main topics; psychoeducation, ADHD strategies for proactive parenting and communication, relationship with child, and attention training. Handouts and resources were provided IY: 12-week, 2–2.5 h sessions: group-based and utilized problem-solving, video modelling, role play, support network and homework to address relationship between parent and child, social, emotional and persistence for language and focus, praise for good behavior, and strategies to manage misbehavior	Cost-effectiveness	Combined societal and National Health Service perspective Estimated the costs to the health service and the family	2013 UK£ No discount 6 months	Swanson Nolan and Pelham (SNAP)-IV–Parent and teacher scales Eyberg Child Behavior Inventory (ECBI) Directly Observed Attention (DOA) Client Service Receipt Inventory (CSRI) General Health Questionnaire (GHQ)	Non recurrent (i.e., course fees/training) Recurrent (i.e., materials, supervision, administration, parent travel costs) Indirect costs (i.e., health services)	The overall total costs for the NFPP were £213,286 ($413,060), and £275,492 ($533,531) for IY	The overall mean total cost was significantly lower for NFPP compared to IY (£1591 vs £2103) ($ 3081 vs $4073), a difference of £512 ($992) (95% CI £324–£700) ($627–$1356) per family Therapist travel costs were higher for NFPP, facility and supervision costs were higher for IY. NFPP and IY did not differ regarding effects on ADHD or conduct problems. However, in the trial context, NFPP was less costly than IY
Tran et al. (2018)	United States	196 parents and children (aged 7–11 years old) CLAS (*n* = 73) PFT (*n* = 74)	Treatment as usual (*n* = 49)	Psychosocial programs	Childhood Life and Attention Skills (CLAS) Program: psychosocial treatment, included home and school components (parent, teacher, and child) Parent-focused treatment (PFT) based on the parent component from the CLAS program	Cost-effectiveness	US modified societal perspective Implementation and parent time costs	2013 US$ No discount 13 weeks (about 3 months)	DSM-IV inattentive symptoms on the Child Symptom Inventory (CSI)	Clinician time, parent time, supplies, coordination and supervision, teacher time, childcare costs	The total (base) cost of PFT and CLAS were $52,561 ($69,680) for 74 patients and $113,813 ($150,880) for 73 patients, respectively	The CLAS intervention had the highest cost, and highest number of resolved ADHD-I cases at post-treatment. An incremental cost per patient of $710 ($941) for PFT and $1559 ($2067) for CLAS when compared to TAU. The incremental cost-effectiveness ratios (ICER) per case resolved were $3997 ($5299) for CLAS versus TAU, $3227 ($4278) for PFT versus TAU, and $4994 ($6620) for CLAS versus PFT. In the initial analysis, the PFT was more cost-effective than CLAS. However, CLAS may be comparably cost-effective by streamlining the model which showed an ICER of $29 ($38)

*DALYs* disability adjusted life years; *HRQoL* health related quality of life; *QALYs* quality-adjusted life years

^a^2024 purchasing power parity PPP estimates international dollars. Costs were converted to 2024 purchasing power parity PPP estimates using the web-based Campbell & Cochrane Economics Methods Group and Evidence for Policy and Practice Information Centre Cost Converter based on Purchasing Power Parity Shemilt et al., 2010. For studies which presented economic data in USD, and not in the country’s local currency, the average exchange rate [https://www.oanda.com/currency-converter/en/] was used to convert the amount to the reported year, before calculating the PPP estimate Seuring et al., 2015

Nystrand et al., [63] found four different behavioral programs (i.e., COPE, Comet, Connect, Incredible Years (IY)) and one resource (Bibliotherapy) all to be cost-effective at $17,600 willingness-to-pay threshold per Disability-Adjusted Life Year (DALY), positioning them as favorable option within standard public health cost-effectiveness thresholds for mental health interventions for awareness and knowledge. The yearly intervention costs ranged from $16 to $1527 per child. Bibliotherapy ($16), COPE ($392) and Connect ($524) dominated the comparator (demonstrated lower accumulated net costs, and greater health benefits (averted DALYs)), however, the IY ($1528) program had the highest effect on averted DALYs (0.23 DALYs per child), and externalizing problems compared to the other programs.

Sayal et al. [64] found that similar behavior programs focusing on behavioral management were likely to be cost-effective, although no significant effects were found on outcomes in this small sample. Intervention costs per participant for the parent-only and combined (parent-teacher) intervention were $178 and $211, respectively. The parent-only intervention was more likely to be cost-effective (with a larger sample) above a willingness-to-pay threshold of $61 per one-point improvement in the ADHD index (Conners’ Parent and Teacher Rating Scale). Below a threshold of $61, neither program was more likely to be cost-effective than treatment as usual [64].

Sonuga-Barke et al. [65] compared the efficacy and cost-effectiveness of a one-on-one (New Forest Parenting Program) versus group parent training (Incredible Years) and found no difference in clinical effectiveness (parent-rated ADHD symptoms) but significantly lower cost per family ($3081 versus $4072) over the six-month evaluation period. Another study compared two psychosocial programs (Child Life and Attention Skills (CLAS), and parent-focused treatment (PFT)) to treatment as usual. Intervention cost per participant were $2066 (CLAS) and $941 (PFT) [66]. The ICER per resolved ADHD case was $5298 (CLAS), $4277 (PFT), and $6620 (CLAS versus PFT). Based on the initial cost effectiveness analysis, the PFT is the most cost-effective option.

## Discussion

### Key findings

The results from this systematic review demonstrate that interventions can be effective in increasing the awareness and knowledge of ADHD among caregivers, clinicians, and teachers. While there was no economic evaluation of interventions with the primary outcome of increasing knowledge, knowledge and skill improvement resulting from these interventions enabled caregivers to manage their child’s ADHD symptoms more positively, such as behavioral management and psychosocial programs. Previous reviews [9, 10] indicate that psychoeducation and other interventions such as behavioral parent training and parent education are effective in increasing awareness and knowledge of ADHD among caregivers. Behavioral management interventions were found to be cost-effective, with costs per participant ranging from $178 to $211 depending on program intensity and delivery format. Similarly, psychosocial programs were also found to be cost effective depending on the delivery mode. Our review contributes to the growing evidence of interventions to increase awareness and knowledge about health conditions [67, 68]. Findings should be interpreted cautiously due to substantial heterogeneity across intervention design, duration, and outcome measurement tools, which may have contributed to various intervention effectiveness and limited cross-study comparability, preventing meta-analysis from being conducted. This heterogeneity made it difficult to draw definitive conclusions about which interventions may be more beneficial, or superior to others in improving ADHD knowledge among caregivers, clinicians, and teachers. Further, we found differences in quality assessment tools whereby the MMAT [33] tended to result in higher quality scores than the NHLBI tools [27]. The difference in overall quality rating between the tools may be explained by the focus of the NHLBI checklists on the internal validity of a study, and whereby the included studies’ risk of bias due to methodological limitations was frequent; poor reporting of randomization techniques, lack of blinding, poor reporting of sample size power and loss to follow-up [37].

The present study’s findings are consistent with the findings from Ward et al. [20] whereby teacher training interventions for ADHD can improve knowledge and positive behaviors toward children with ADHD symptoms. Our review advanced the previous review by including ten studies on other teacher populations, such as graduate and pre-service teachers. Our findings demonstrate that teachers’ knowledge and awareness of ADHD including knowledge and practice of behavioral management skills, and ability to detect ADHD symptoms in children were improved with knowledge training/programs consisting of lectures, webinars and workshops supported by roleplay, discussion, and handouts. While this review found the teacher-focused training interventions are effective, ADHD knowledge among teachers may not have significantly improved over time [7]. There is a need for ongoing teacher training interventions such as the programs discussed in this review, as poor ADHD knowledge is still reported as a key barrier to diagnosing and managing the condition in children [4–6].

Our review demonstrates that training and educational interventions can be effective in increasing ADHD knowledge among caregivers. Specific knowledge and skill development to inform parents about ADHD and supporting their child is important for improving parental recognition of symptoms, reducing stigma, misconceptions, in turn increasing caregivers’ willingness access and utilize healthcare services [11, 69].

We found that there is little evidence to preference one program over another. For example, a multi-component intervention did not have greater effects than a parent education only program [70], and a group intervention format was not inferior to individual behavioral parenting training (BPT) [71]. It is likely that patient preferences of format (online versus in-person, clinic versus home-based) and the intervention costs are the most prominent factors to consider when delivering interventions [72]. Group-based behavioral parent training has previously been recommended for pre-school aged children [73] as it is equally effective and can be more cost-effective than individual-based training. The common pattern across the heterogeneous interventions is that most have resulted in increases in knowledge and capacity, demonstrating that positive outcomes can be achieved through lower-resource-required interventions such as single or few session webinars, or workshops.

To our knowledge, this is the first review to synthesize evidence on interventions to address clinician ADHD awareness and knowledge. We found that clinician-targeted interventions were received well, with participants highlighting formats as appropriate, resources being useful and addressed ‘some to most’ of general practitioner’s knowledge needs [49, 50]. Depending on intervention types, our review found that clinician awareness and knowledge of ADHD and their self-efficacy in integrating evidence-based ADHD practices for diagnosis and treatment can be effectively increased in the short term through the delivery of a 40-min web-based resource [49], a 1 day course [14], or can be sustained for up to 24 months when the intervention involves continuous quality improvement focused on aligning clinician practices with the American Academy of Pediatrics consensus guideline recommendations [48]. Although clinicians’ and healthcare professionals’ ADHD awareness and knowledge have improved over time, the evolving evidence on diagnosis and treatment approaches requires a frequent update of knowledge, and thus, ongoing interventions would be needed to upskill clinicians in ADHD management, including knowledge of care pathways, diagnosis and treatment [74, 75].

### Economic evaluation

This current review is the first to synthesize economic evidence on interventions that have included an ADHD education component for caregivers and teachers. Whilst none of the interventions directly measured knowledge as a primary outcome, ADHD educational/knowledge component was embedded in the intervention content. Parent and teacher knowledge and skill improvement resulting from these interventions could have enabled caregivers and teachers to support children with ADHD more positively. We found that these interventions led to improved outcomes of averted DALYs and resolved ADHD cases. All these four studies found that interventions with educational components were promising or cost-effective, depending on the intensity and the format/design (Table 2). Behavioral interventions focusing on behavioral management were found to be cost-effective and effective in reducing DALYs [63]. Ongoing economic evaluation of interventions, including for caregivers, clinicians, and teachers is crucial as the information on costs and cost-effectiveness can assist policy-making decisions on population health resources allocation [72].

### Implications for policy and practice

Lack of ADHD awareness and knowledge among caregivers, clinicians, and teachers have long been key barriers to service access and utilization [4, 6, 76], therefore, promoting these interventions to improve awareness and knowledge of ADHD are important to enable timely access and utilization of services for diagnosis and treatment. We support the recommendation in the Australian clinical guidelines which encourages the provision of education and training to clinicians to improve their knowledge and capacity for evidence-based practices, to reduce stigma and misconceptions that can act as barriers to service utilization [13].

Interventions can demonstrate modest improvements at relatively low cost, or substantial improvements and higher costs, however, the limited economic evidence in our review suggests they offer quite similar overall returns on investment and therefore, all could be considered depending on health system priority areas and available resources/allocated budget.

The scarcity of literature on economic evaluation of ADHD interventions targeted at caregivers, clinicians, and teachers highlights a need for more cost-effectiveness studies of interventions among these populations. Given the high heterogeneity in outcome measures in effectiveness studies, it would be beneficial for future research to explore a common core outcome set that can be applied to all ADHD interventions aiming to improve ADHD awareness/knowledge to enable cross-study outcome comparison.

### Limitations

Restricting the inclusion criteria to peer-reviewed and English language may have resulted in missing unpublished or grey literature, or non-English literature containing evidence on the effectiveness and cost-effectiveness of interventions. Excluding non-English studies could introduce bias omitting studies from regions where interventions may have been evaluated differently. The search term for economic evaluation used for the Google Scholar search was cost-effectiveness, and therefore may have resulted in missing relevant studies. Studies in this review demonstrate a limitation in the lack of consistency across interventions and outcome measures (high heterogeneity) which prevented comparability across studies.

## Conclusion

This review is the first to explore the effectiveness and cost-effectiveness of interventions on ADHD awareness/knowledge and management among caregivers and clinicians and updated the existing synthesis of the literature on teacher ADHD training interventions. We found that behavioral interventions and psychosocial programs are not only cost-effective but also support a structured approach to enhancing ADHD knowledge across parents and teachers, adding significant depth to the current understanding of ADHD awareness and intervention impacts. The review highlights a need for ongoing training for caregivers, clinicians, and teachers. Further research in economic evaluation of interventions is recommended to assist policy decision-making.

## Supplementary Information

Below is the link to the electronic supplementary material.

Supplementary file1 (DOCX 33 KB)

## Data Availability

No datasets were generated or analysed during the current study.
