# Supplementary material for: Effectiveness and cost-effectiveness of interventions to increase knowledge and awareness of attention deficit hyperactivity disorder: a systematic review
Source: Eur Child Adolesc Psychiatry. 2025 Jan 26;34(6):1769–94. doi: 10.1007/s00787-025-02646-4 (PMC12198261; doi:10.1007/s00787-025-02646-4)
Supplement: Supplementary file 1 — Supplementary file1 (DOCX 33 KB) [file 787_2025_2646_MOESM1_ESM.docx]

**Appendix A: Search strategy**

| Table A1: Search terms used for each database | | |
| --- | --- | --- |
| 1 | “ADHD” OR “attention deficit hyperactivity disorder*” OR "attention deficit disorder*" OR “attention-deficit/hyperactivity disorder” OR “attention-deficit hyperactivity disorder” OR “attention-deficit-hyperactivity disorder” OR (MH "Attention Deficit Disorder with Hyperactivity") | TI OR AB |
| 2 | cost* OR invest* OR “return-on-investment” OR econom* OR financ* OR expenditure* OR spend* OR expense* OR “net benefit*” OR “cost-utility” OR “cost-benefit” OR “cost-effectiveness” OR “value-for-money” OR “value for money” OR (MH "Cost-Benefit Analysis") OR (MH “Cost Savings”) OR (MH "Cost-Effectiveness Analysis") OR (MH "Costs and Cost Analysis+") OR effect* OR efficac* OR impact* OR evaluat* | TI OR AB |
| 3 | (intervent* OR program* OR train* OR promot* OR support* OR initiative* OR approach* OR technique* OR strateg* OR assess* OR service* OR coach* OR chang*) N5 (aware* OR knowledg* OR understand* OR educat* OR attitud* OR belie* OR learn* OR literacy OR inform* OR train* OR recogni* OR acknowledg*) | TI OR AB |
| 4 | 1 AND 2 AND 3 |  |

TI: Title search, AB: Abstract search

| Table A2: Search terms used Google Scholar search | |
| --- | --- |
| 1 | ADHD |
| 2 | Education |
| 3 | Cost-effectiveness |

**Appendix B: Quality assessment tools and scoring technique**

The scoring technique is described in each quality assessment table. For the NHLBI controlled intervention studies, and before-after (pre-post) studies with no control group, studies were considered as ‘good’, ‘fair,’ and ‘poor’ if at least 80%, 51-71% and 50% (less than or equal to) of criteria were met, respectively (Hirsch, van Wegen, Newman, & Heyn, 2018). Quality assessment of cross-sectional studies used only the applicable questions of the tool, which were criteria 1-6, and 11 (Gundmi et al., 2018; Wanni Arachchige Dona et al., 2023).
For quantitative non-randomized studies using the Mixed Methods Appraisal Tool (Hong et al., 2018), overall quality was rated as low (1-2), medium (3), or high (4-5) based on the total score (Evangelio, Rodriguez-Gonzalez, Fernandez-Rio, & Gonzalez-Villora, 2022).
Drummond’s 35-item checklist (Drummond & Jefferson, 1996) was used to assess the quality of economic evaluation studies as recommended by the Cochrane Collaboration (Higgins, 2008). If the % was >75%, the study quality was considered good (Donker et al., 2015; Feliu-Soler et al., 2018), however, studies were not categorized from low to high quality as it is largely accepted that the higher the number of criteria met, the higher the study quality.

**Appendix C: Quality assessment outcome**

Appendix C1: NHLBI Quality Assessment of Controlled Intervention Studies

| Study | Q1 | Q2 | Q3 | Q4 | Q5 | Q6 | Q7 | Q8 | Q9 | Q10 | Q11 | Q12 | Q13 | Q14 | T | QR |
| --- | --- | --- | --- | --- | --- | --- | --- | --- | --- | --- | --- | --- | --- | --- | --- | --- |
| (Alshehri et al., 2020) | Y | Y | Y | N | N | Y | Y | Y | Y | Y | Y | Y | Y | Y | 12 | G |
| (DuPaul et al., 2018) | Y | CD | CD | N | N | Y | Y | Y | Y | NR | Y | N | Y | CD | 7 | P |
| (French et al., 2020) | Y | Y | Y | Y | N | N | N | Y | Y | NR | Y | CD | Y | N | 8 | F |
| (Gerdes et al., 2021) | Y | CD | CD | NR | NR | Y | Y | Y | Y | NR | Y | N | Y | Y | 8 | F |
| (Jones & Chronis-Tuscano, 2008) | Y | CD | CD | NR | NR | Y | Y | Y | Y | NR | Y | NR | Y | Y | 8 | F |
| (Odom, 1996) | Y | CD | CD | N | NR | Y | Y | Y | Y | NR | Y | NR | Y | N | 7 | P |
| (Jimenez et al., 2022) | Y | CD | CD | NR | NR | Y | Y | CD | Y | NR | Y | NR | Y | Y | 7 | P |
| (White et al., 2011) | Y | CD | CD | NR | NR | NR | N | CD | Y | NR | Y | N | NR | Y | 4 | P |
| For each item: Yes (Y) = 1, No (N) = 0, Cannot Determine (CD) = 0, Not Reported (NR) = 0, Not Applicable (NA) = 0. Total score (T).  Overall quality rating (QR) : Good (G) = >80%, Fair (F) = 51-71%, Poor (P) = 50% or less. <https://www.nhlbi.nih.gov/health-topics/study-quality-assessment-tools> | | | | | | | | | | | | | | | |  |

Appendix C2: NHLBI Quality Assessment Tool for Before-After (Pre-Post) Studies With No Control Group

| Study | Q1 | Q2 | Q3 | Q4 | Q5 | Q6 | Q7 | Q8 | Q9 | Q10 | Q11 | Q12 | T | QR |
| --- | --- | --- | --- | --- | --- | --- | --- | --- | --- | --- | --- | --- | --- | --- |
| (Baum et al., 2019) | Y | Y | Y | Y | NR | Y | Y | NR | Y | Y | Y | Y | 10 | G |
| (Bradley-Klug et al., 1997) | Y | Y | Y | NR | NR | Y | Y | NR | N | Y | Y | Y | 8 | F |
| (Dixon et al., 2023) | Y | Y | Y | Y | N | Y | Y | NR | N | Y | N | NA | 7 | F |
| (Epstein et al., 2010) | Y | Y | Y | Y | N | Y | Y | NR | N | Y | N | NA | 7 | F |
| (Giannopoulou et al., 2017) | Y | Y | Y | Y | N | Y | Y | NR | Y | Y | N | NA | 8 | F |
| (Loskutova et al., 2021) | Y | Y | Y | NR | NR | Y | Y | NR | Y | Y | Y | NA | 8 | F |
| (Monteiro, 2023) | Y | Y | Y | NR | Y | Y | Y | NR | NR | Y | N | NA | 7 | F |
| (Newcomb et al., 2022) | Y | Y | Y | Y | NR | Y | Y | NR | N | Y | N | NA | 7 | F |
| (Pfiffner et al., 2023) | Y | Y | N | Y | NR | Y | Y | NR | NR | Y | N | NA | 6 | P |
| (Ryan et al., 2015) | Y | Y | Y | Y | Y | Y | Y | NR | Y | Y | N | NA | 9 | F |
| (Sayal et al., 2006) | Y | Y | Y | Y | Y | Y | Y | NR | Y | Y | N | NA | 9 | F |
| (Shata et al., 2014) | Y | Y | Y | Y | NR | Y | Y | NR | Y | Y | Y | NA | 9 | F |
| (R. Ward et al., 1999) | Y | Y | Y | NR | NR | Y | Y | NR | N | Y | N | NA | 6 | P |
| For each item: Yes (Y) = 1, No (N) = 0, Cannot Determine (CD) = 0, Not Reported (NR) = 0, Not Applicable (NA) = 0. Total score (T). Overall quality rating (QR): Good (G) = >80%, Fair (F) = 51-71%, Poor (P) = 50% or less. <https://www.nhlbi.nih.gov/health-topics/study-quality-assessment-tools> | | | | | | | | | | | | | |  |

Appendix C3: NHLBI Quality Assessment Tool for Observational Cohort and Cross-Sectional Studies

| Study | Q1 | Q2 | Q3 | Q4 | Q5 | Q11 | T | QR |
| --- | --- | --- | --- | --- | --- | --- | --- | --- |
| (Awadalla et al., 2016) | Y | Y | Y | Y | Y | Y | 6 | G |
| For each item: Yes (Y) = 1, No (N) = 0, Cannot Determine (CD) = 0, Not Reported (NR) = 0, Not Applicable (NA) = 0. Total score (T). Overall quality rating (QR): Good (G) = 6, Fair (F) = 4-5 Poor (P) = <4. <https://www.nhlbi.nih.gov/health-topics/study-quality-assessment-tools> | | | | | | | | |

Appendix C4: Mixed Methods Appraisal Tool 3. Quantitative Non-Randomized Studies

| Study | Q3.1 | Q3.2 | Q3.3 | Q3.4 | Q3.5 | T | QR |
| --- | --- | --- | --- | --- | --- | --- | --- |
| (Jordan et al., 2004) | Y | Y | N | N | Y | 3 | M |
| (Srivastava et al., 2015) | Y | Y | Y | Y | Y | 5 | H |
| For each item: Yes (Y) = 1, No (N) = 0, Cannot tell (CT) = 0.5. Total score (T). Overall quality rating (QR): Low (L) = 1–2, Medium (M) = 3, High (H) = 4-5. | | | | | | | |

Appendix C5: Drummond Checklist: 35 item

| Study | Q1 | Q2 | Q3 | Q4 | Q5 | Q6 | Q7 | Q8 | Q9 | Q10 | Q11 | Q12 | Q13 | Q14 | Q15 | Q16 | Q17 | Q18 |  |
| --- | --- | --- | --- | --- | --- | --- | --- | --- | --- | --- | --- | --- | --- | --- | --- | --- | --- | --- | --- |
| (Nystrand et al., 2019) | Y | Y | Y | Y | Y | Y | Y | Y | Y | NA | Y | Y | Y | NA | NA | N | Y | Y |  |
| (Sayal et al., 2016) | Y | Y | Y | Y | Y | Y | Y | Y | Y | NA | Y | Y | Y | N | N | N | Y | Y |  |
| (Sonuga-Barke et al., 2018) | Y | Y | Y | Y | Y | Y | Y | Y | Y | NA | Y | NA | NA | NA | NA | N | Y | Y |  |
| (Tran et al., 2018) | Y | Y | Y | Y | Y | Y | Y | Y | Y | NA | Y | Y | Y | Y | Y | Y | Y | Y |  |
|  |  |  |  |  |  |  |  |  |  |  |  |  |  |  |  |  |  |  |  |
| Study continued. | Q19 | Q20 | Q21 | Q22 | Q23 | Q24 | Q25 | Q26 | Q27 | Q28 | Q29 | Q30 | Q31 | Q32 | Q33 | Q34 | Q35 | T | QR |
| (Nystrand et al., 2019) | Y | Y | Y | Y | Y | Y | NA | Y | Y | Y | Y | Y | Y | N | Y | Y | Y | 93.5% | G |
| (Sayal et al., 2016) | N | Y | Y | Y | NA | NA | N | Y | N | NA | NA | Y | Y | N | Y | Y | Y | 76.6% | G |
| (Sonuga-Barke et al., 2018) | N | Y | Y | Y | NA | NA | N | Y | Y | N | N | Y | N | N | NC | Y | Y | 71.4% | - |
| (Tran et al., 2018) | N | Y | NC | Y | NA | NA | N | Y | Y | Y | N | Y | Y | N | Y | Y | Y | 84.4% | G |
| For each item: Yes (Y) = 1, No (N) = 0, Not Clear (NC) = 0, Not Appropriate (NA) = 0. Total score (T). Overall quality rating (QR). | | | | | | | | | | | | | | | | | | |  |
